# Detection of *TP53* Mutations in Tissue or Liquid Rebiopsies at Progression Identifies ALK^+^ Lung Cancer Patients with Poor Survival

**DOI:** 10.3390/cancers11010124

**Published:** 2019-01-21

**Authors:** Petros Christopoulos, Steffen Dietz, Martina Kirchner, Anna-Lena Volckmar, Volker Endris, Olaf Neumann, Simon Ogrodnik, Claus-Peter Heussel, Felix J. Herth, Martin Eichhorn, Michael Meister, Jan Budczies, Michael Allgäuer, Jonas Leichsenring, Tomasz Zemojtel, Helge Bischoff, Peter Schirmacher, Michael Thomas, Holger Sültmann, Albrecht Stenzinger

**Affiliations:** 1 Department of Thoracic Oncology, Heidelberg University Hospital, 69126 Heidelberg, Germany; petros.christopoulos@med.uni-heidelberg.de (P.C.); helge.bischoff@med.uni-heidelberg.de (H.B.); michael.thomas@med.uni-heidelberg.de (M.T.); 2 Translational Lung Research Center Heidelberg (TLRC-H), Member of the German Center for Lung Research (DZL), 69120 Heidelberg, Germany; s.dietz@dkfz-heidelberg.de (S.D.); heussel@uni-heidelberg.de (C.-P.H.); felix.herth@med.uni-heidelberg.de (F.J.H.); martin.eichhorn@med.uni-heidelberg.de (M.E.); michael.meister@med.uni-heidelberg.de (M.M.); 3 Division of Cancer Genome Research, German Cancer Research Center (DKFZ) and National Center for Tumor Diseases (NCT), 69120 Heidelberg, Germany; s.ogrodnik@dkfz-heidelberg.de; 4 German Cancer Consortium (DKTK), 69120 Heidelberg, Germany; jan.budczies@med.uni-heidelberg.de; 5 Institute of Pathology, Heidelberg University Hospital, 69120 Heidelberg, Germany; Martina.Kirchner@med.uni-heidelberg.de (M.K.); anna-lena.volckmar@med.uni-heidelberg.de (A.-L.V.); volker.endris@med.uni-heidelberg.de (V.E.); olaf.neumann@med.uni-heidelberg.de (O.N.); michael.allgaeuer@med.uni-heidelberg.de (M.A.); jonas.leichsenring@med.uni-heidelberg.de (J.L.); peter.schirmacher@med.uni-heidelberg.de (P.S.); 6 Diagnostic and Interventional Radiology with Nuclear Medicine, Thoraxklinik at Heidelberg University Hospital, 69126 Heidelberg, Germany; 7 Department of Diagnostic and Interventional Radiology, Heidelberg University Hospital, 69120 Heidelberg, Germany; 8 Department of Pneumology, Thoraxklinik at Heidelberg University Hospital, 69126 Heidelberg, Germany; 9 Department of Surgery, Thoraxklinik at Heidelberg University Hospital, 69126 Heidelberg, Germany; 10 Translational Research Unit, Thoraxklinik at Heidelberg University Hospital, 69126 Heidelberg, Germany; 11 BIH-Genomics Core Unit, Charité-Universitätsmedizin Berlin, 13125 Berlin, Germany; tomasz.zemojtel@bihealth.de

**Keywords:** anaplastic lymphoma kinase positive (ALK^+^) non-small cell lung cancer (NSCLC), tumor protein p53 gene (*TP53*) mutation, tyrosine kinase inhibitor, progression-free survival, overall survival

## Abstract

Anaplastic lymphoma kinase (ALK) sequencing can identify resistance mechanisms and guide next-line therapy in ALK^+^ non-small-cell lung cancer (NSCLC), but the clinical significance of other rebiopsy findings remains unclear. We analysed all stage-IV ALK^+^ NSCLC patients with longitudinally assessable *TP53* status treated in our institutions (*n* = 62). Patients with *TP53* mutations at baseline (*TP53*mut^bas^, *n* = 23) had worse overall survival (OS) than patients with initially wild-type tumours (*TP53*wt^bas^, *n* = 39, 44 vs. 62 months in median, *p* = 0.018). Within the generally favourable *TP53*wt^bas^ group, detection of *TP53* mutations at progression defined a “converted” subgroup (*TP53*mut^conv^, *n* = 9) with inferior OS, similar to that of *TP53*mut^bas^ and shorter than that of patients remaining *TP53* wild-type (*TP53*wt^progr^, 45 vs. 94 months, *p* = 0.043). Progression-free survival (PFS) under treatment with tyrosine kinase inhibitors (TKI) for *TP53*mut^conv^ was comparable to that of *TP53*mut^bas^ and also shorter than that of *TP53*wt^progr^ cases (5 and 8 vs. 13 months, *p* = 0.0039). Fewer *TP53*wt^progr^ than *TP53*mut^bas^ or *TP53*mut^conv^ cases presented with metastatic disease at diagnosis (67% vs. 91% or 100%, *p* < 0.05). Thus, acquisition of *TP53* mutations at progression is associated with more aggressive disease, shorter TKI responses and inferior OS in ALK^+^ NSCLC, comparable to primary *TP53* mutated cases.

## 1. Introduction

Anaplastic lymphoma kinase (*ALK*) gene fusions are driver genetic alterations in approximately 5% of non-small-cell lung cancers (NSCLC) [1]. Α breakthrough in their treatment was the development of several ALK tyrosine kinase inhibitors (TKI), which in sequential administration have pushed median patient survival to over five years [2]. Analysis of TKI failure has therefore become a main focus of research efforts, because its prediction, mechanistic dissection and individualized treatment is of key importance for further therapeutic advances.

Recent studies combining state-of-the-art molecular profiling with detailed clinical annotation have identified two molecular risk factors associated with TKI failure in ALK-driven NSCLC: echinoderm microtubule-associated protein-like 4 (*EML4*)*-ALK* fusion variant V3 [3,4,5] and the presence of tumor protein p53 gene (*TP53*) mutations at initial diagnosis [6,7,8]. They occur independent from each other in about 30–40% and 20% of patients, respectively, have synergistic effects and are both associated with shorter progression-free survival (PFS) after treatment with first- and second-generation ALK TKI and with worse overall survival (OS) [8].

Furthermore, molecular workup of a follow-up tissue or liquid biopsy at the time of TKI failure is gaining importance for the management of ALK^+^ NSCLC, since it can reveal patient-specific resistance mechanisms and guide subsequent therapeutic decisions [9,10,11]. In particular, detection of *ALK* resistance mutations can be useful for the selection of suitable next-line TKI based on in vitro sensitivity data [12]. However, the clinical importance of other molecular findings in tumor rebiopsies remains unclear.

Here, we examine the significance of *TP53* mutations detected at the time of disease progression in ALK^+^ NSCLC patients with *TP53* wild-type status at initial diagnosis.

## 2. Results

**Figure 1:**
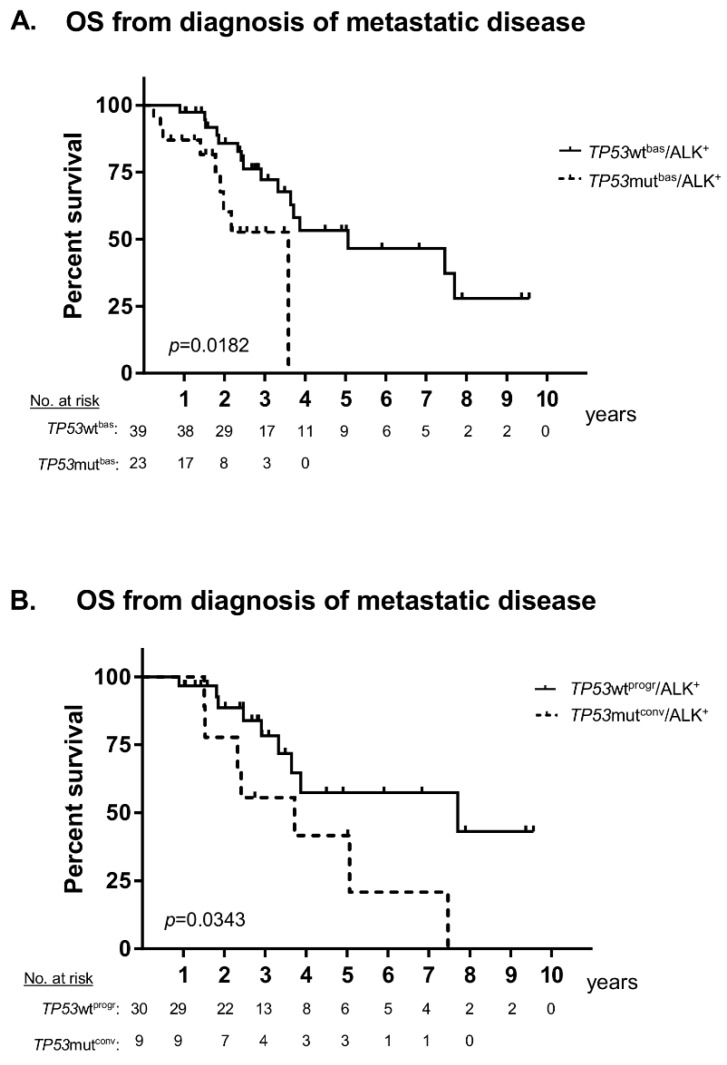
Overall survival of patients with metastatic anaplastic lymphoma kinase-positive (ALK^+^) non-small cell lung cancer (NSCLC) according to *TP53* status at baseline and progression. (**A**) The median overall survival (OS) was 44 months for patients with *TP53* mutations at baseline (*TP53*mutbas) vs. 62 months for patients without *TP53* mutations at baseline (*TP53*wtbas logrank *p* = 0.0182). (**B**) The median OS was 45 months for patients with initially wild-type status and detection of *TP53* mutations in a subsequent biopsy (*TP53*mutconv) vs. 94 months for patients without subsequent detection of *TP53* mutations (*TP53*wtprogr, logrank *p* = 0.0343). Treatment details are given in Table 1.

**Figure 2:**
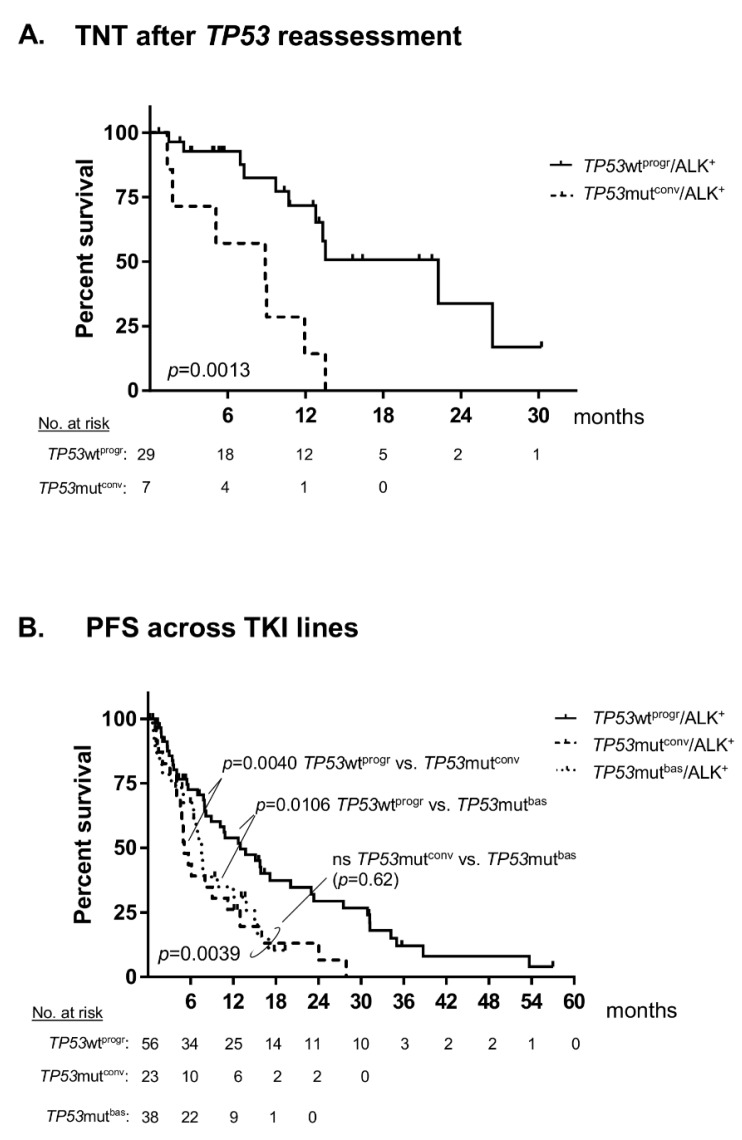
Progression-free survival (PFS) of patients with metastatic anaplastic lymphoma kinase-positive (ALK^+^) non-small cell lung cancer (NSCLC) under treatment with tyrosine kinase inhibitors (TKI) according to *TP53* status at baseline and progression. (**A**) The median time-to-next-treatment (TNT) under TKI for patients with initially wild-type *TP53* tumours after reassessment of *TP53* status was 9 months for cases with a positive result (*TP53*mut^conv^) vs. 23 months for cases with negative result (*TP53*wt^progr^, logrank *p* = 0.0013). Treatment details including continuation of treatment beyond disease progression are given in Table 1. (**B**) The median PFS under TKI treatment across all treatment lines was 8 months for patients with *TP53* mutations at baseline (*TP53*mut^bas^) vs. 5 months for patients with initially wild-type status and detection of *TP53* mutations in a subsequent biopsy (*TP53*mut^conv^) vs. 13 months for patients without subsequent detection of *TP53* mutations (*TP53*wt^progr^, logrank *p* = 0.0039); ns: not significant.

ALK^+^ NSCLC patients with tumour *TP53* mutations at baseline (*TP53*mut^bas^) had a worse overall survival (OS) from the diagnosis of metastatic disease than patients with initially wild-type *TP53* tumours (*TP53*wt^bas^, 44 vs. 62 months in median, *p* = 0.0182, Figure 1A). Within the generally favourable *TP53*wt^bas^ patient group, detection of *TP53* mutations in a subsequent tissue or liquid biopsy performed at disease progression identified an unfavourable, *TP53* “converted” patient subgroup (*TP53*mut^conv^, Table 1) with OS comparable to that of *TP53*mut^bas^ patients and shorter than that of patients retaining *TP53* wild-type status after progression (*TP53*wt^progr^, 45 vs. 94 months in median, *p* = 0.0343, Figure 1B). These comprised 23% (9/39) of initially *TP53* wild-type (*TP53*wt^bas^) cases in our cohort. The newly acquired *TP53* mutations resided in exons 5–10 of *TP53* (Supplementary Table S1), i.e., in genetic regions that had already been tested as wild-type at initial diagnosis, because they were included in the NGS panel of both methods used in this study. All of them were pathogenic and resulted in loss-of-function (Supplementary Table S1). The time-to-next-treatment (TNT) for patients treated with TKI after the reassessment of *TP53* status, was significantly shorter for cases with a positive (*TP53*mut^conv^) than for cases with a negative (*TP53*wt^progr^) result (9 vs. 23 months in median, *p* = 0.0013, Figure 2A). In addition, PFS under treatment with ALK TKI across treatment lines for patients with secondary detection of *TP53* mutations at progression (*TP53*mut^conv^) was comparable to that of patients with *TP53* mutations at baseline (*TP53*mut^bas^), and also shorter than that of patients retaining *TP53* wild-type status (*TP53*wt^progr^, 5 and 8 vs. 13 months in median, *p* = 0.0039, Figure 2B). In contrast, there was no significant difference in the PFS under chemotherapy across treatment lines between patients of the three groups according to *TP53* status (7 and 5 vs. 8 months in median, respectively, *p* = 0.60, Supplementary Figure S1).

Analysis of initial clinical presentation revealed that patients retaining *TP53* wild-type status after disease progression (*TP53*wt^progr^) had featured a lower rate of metastatic disease at initial diagnosis than patients with *TP53* mutations either at baseline (*TP53*mut^bas^, 67% vs. 91%, *p* = 0.034, Table 1) or at disease progression (*TP53*mut^conv^, 67% vs. 100%, *p* = 0.045, Table 1). The OS from initial diagnosis was also similar between patients with *TP53* mutations detected either at diagnosis (*TP53*mut^bas^) or at disease progression (*TP53*mut^conv^) and worse than that of patients retaining *TP53* wild-type status (TP53wt^progr^, 44 and 45 months in median vs. not reached, *p* = 0.0012, Supplementary Figure S2).

## 3. Discussion

The results presented here extend the findings of recent studies that demonstrated the major clinical significance of baseline *TP53* mutational status in ALK^+^ NSCLC [6,7,8] As in a previous report [8], presence of *TP53* mutations in our patients at initial diagnosis was associated with shorter PFS under TKI (Figure 2B) and worse OS (Figure 1A), but did not apparently affect benefit from chemotherapy (Supplementary Figure S1).

The main novel finding of this study is that secondary detection of *TP53* mutations at disease progression in patients with wild-type *TP53* at baseline has a similar negative impact. Both PFS under TKI treatment and OS were shorter for initially wild-type patients with *TP53* mutations detected later in the course of the disease (*TP53*mut^conv^), when compared to patients retaining the *TP53* wild-type status (*TP53*wt^ther^, Figure 2B and Figure 1B, respectively). Thus, among the generally favourable group of initially *TP53* wild-type ALK^+^ tumours, acquisition of *TP53* mutations identifies an unfavourable subgroup with a clinical course similar to that of primarily *TP53* mutated cases. Emergence of *TP53* mutations at the time of disease progression was observed in 23% (9/39) of initially *TP53* wild-type patients in our cohort (Table 1). Of note, a similar percentage (20–24%) of metastatic ALK^+^ NSCLC has been reported to harbor *TP53* mutations at baseline in two recent series [7,8], which adds up to *TP53* mutations being detectable in approximately 40–50% of TKI-refractory ALK^+^ NSCLC patients, as had already been noted in an earlier study [12]. It should also be mentioned here, that *TP53* mutations are overrepresented among the baseline samples of the current study, because all cases with detectable *TP53* mutations at diagnosis were included, but several initially *TP53* wild-type tumours had to be excluded, because no *TP53* reassessment was available. Acquisition of *TP53* alterations with disease progression has also been noted in various hematologic malignancies, like chronic lymphocytic leukemia [13] and multiple myeloma [14], in which it is also associated with worse outcome.

The comparable prognostic and predictive role of *TP53* mutations in *TP53*mut^bas^ and *TP53*mut^conv^ ALK^+^ NSCLC suggest a similar adverse biology in these tumours, regardless of the time-point and context of *TP53* mutation detection. One possibility is that other, more basic and still unidentified biologic alterations in *TP53* mutated tumours might exert an even more important influence on clinical course, and that these could be active already before *TP53* mutations become detectable, which may have implications for the ongoing efforts to target mutant *TP53* with novel drugs [15]. At the same time, it cannot be excluded that due to intratumour heterogeneity, a tumour might initially be tested as *TP53* wild-type on a *TP53* wild-type region of the neoplasm, despite having a similar overall *TP53* mutation load as tumours with readily detectable *TP53* mutations. Indeed, analysis of surgical specimens has shown that *TP53* sequencing results can be variable between different regions of the same tumour [16,17].

A noninvasive strategy to overcome the impact of intratumour heterogeneity could be to perform liquid biopsies (ctDNA assays) in addition to tissue biopsies in cases when a more accurate determination of *TP53* status is needed, e.g., for purposes of prospective molecular risk stratification. The similarly adverse role of initially and subsequently detected *TP53* mutations in our patients (Figure 1 and Figure 2), in combination with the predominance of ctDNA over tissue (i.e., FFPE DNA) assays among *TP53* assessments under therapy in our study (Table 1, including footnote 4, and Supplementary Table S1) support the feasibility of this approach. Even though sensitivity of liquid biopsies for the detection of mutations is lower than 100%, for example it was determined as 60–70% regarding *EGFR* T790M in a recent study [18], we detected a *TP53* mutation in the baseline ctDNA sample of a patient with wild-type *TP53* in the respective biopsy (Supplementary Table S1), but cannot estimate the frequency of this constellation, because we lack baseline ctDNA samples for the majority of our patients.

In summary, the results of this study extend the picture of adverse clinical outcome associated with *TP53* mutations in ALK^+^ NSCLC, and demonstrate the great potential of ctDNA assays for molecular profiling and longitudinal monitoring in ALK^+^ NSCLC beyond detection of *ALK* resistance mutations.

## 4. Materials and Methods

This study included all patients treated at our institution for histologically confirmed, ALK-driven NSCLC with *TP53* status assessment at baseline and/or after disease progression after informed consent and approval by the Heidelberg University ethics committee (S-296/2016). Characteristics of study patients are summarized in Table 1. Biosamples were provided by BioMaterial Bank Heidelberg (BMBH) in accordance with its regulations and after approval by the Heidelberg University ethics committee. Clinical data were collected through a review of patient records and radiological images with chest CT and brain MRI-based restaging every 6–12 weeks. PFS was evaluated according to RECIST v1.1 [19]. The presence of an *ALK* translocation was ascertained by positive results in at least two of the following assays: ALK immunohistochemistry (D5F3 clone, Roche, Mannheim, Germany), *ALK* fluorescent in situ hybridisation (ZytoLight SPEC ALK probe, ZytoVision, Bremerhaven, Germany) and RNA-based next-generation sequencing (NGS, ThermoFisher Lung Cancer Fusion Panel, Waltham, MA, USA), as published previously (details are given in the Supplements) [5,20,21]. *TP53* status was determined either on formalin-fixed paraffin-embedded (FFPE) tissue samples by DNA-based NGS using a proprietary Lung Cancer Panel that covers the entire *TP53* exons 4, 5, 6, 7, 8, 9, 10, as published previously [8,21,22], and/or by plasma DNA genotyping using the AVENIO ctDNA Targeted kit that covers the entire *TP53* exons 2, 3, 4, 5, 6, 7, 8, 9, 10, 11, according to the manufacturer’s instructions (Roche, Mannheim, Germany; details are given in the Supplementary Files under supplementary Methods S1–S4). Baseline *TP53* status was either directly determined by analysis of tumour samples obtained before treatment start in 51/62 cases or inferred as wild-type based on a negative *TP53* result in an assessment performed at the time of disease progression in the remaining cases (Table 1 and Supplementary Table S1). *TP53* status under therapy was determined by analysing tissue and/or blood (ctDNA) samples obtained after disease progression (Table 1 and Supplementary Table S1). Patients with detection of *TP53* mutations at disease progression, but unknown baseline status, as well as patients with wild-type baseline *TP53* status without reassessment after progression, were excluded from this analysis. In contrast, all patients with *TP53* mutations at baseline were included. Survival data were analysed according to Kaplan–Meier and compared between patient subgroups with the logrank test. Median follow-up time was calculated by the reverse Kaplan–Meier method. Categorical data were compared with the chi-square test. Statistical calculations were performed with SPSS version 24 (IBM, Armonk, NY, USA) and plots generated with GraphPad Prism version 7 (GraphPad Software, La Jolla, CA, USA).

## 5. Conclusions

This study shows that detection of *TP53* mutations in tissue of liquid rebiopsies at the time of disease progression in previously negative patients is associated with more aggressive clinical course, shorter TKI responses and inferior OS in ALK^+^ NSCLC, comparable to primary *TP53* mutated cases. These results extend the picture of adverse clinical outcome associated with *TP53* mutations in ALK^+^ NSCLC, and demonstrate the great potential of ctDNA assays for molecular profiling and longitudinal monitoring in ALK^+^ NSCLC beyond detection of *ALK* resistance mutations.

## Figures and Tables

**Table 1:** Patient characteristics and details of treatment.

All Study Patients (*N* = 62)		*TP53*wt^bas^ (*n* = 39)		*TP53*mut^bas^ (*n* = 23)
		*TP53*wt^progr^ (*n* = 30)	*TP53*mut^conv^ (*n* = 9)	
Age (median; IQR)		51; 17	63; 20	65; 19
Sex (male/female)		15/16	5/4	12/11
ECOG PS at diagnosis of stage IV (median; IQR)		0; 0	0; 0	1; 0
Histology	adenocarcinoma ^1^	29/30	9/9	23/23
ALK status	positive	all cases by inclusion criteria		
	*EML4-ALK* V3 ^2^	8/24	5/9	8/20
Stage IV NSCLC	at initial diagnosis	20/30 *	9/9	21/23
	M1a	7/20	1/9	5/21
	by relapse of M0 NSCLC	10/30	0/9	2/23
*TP53* assessment at baseline + at progression ^3^				
method	FFPE at BL +FFPE at PD ^4^	8/30 (neg + neg)	2/9 (neg + pos)	See Table S1
	FFPE at BL +ctDNA at PD	11/30 (neg + neg)	7/9 (neg + pos)	
	FFPE at PD ^4^	6/30 (neg)		
	only ctDNA at PD	5/30 (neg)		
TKI line (start) at 2nd assessment (median; IQR)		2; 1	2; 1	
treatment line at 2nd assessment (median; IQR)		2; 3	4; 1	
- days after diagnosis of stage IV (median; IQR)		702; 1056	752; 600	
ALK TKI treatment, next-line				
	crizotinib	14	2	
	ceritinib	7	4	
	alectinib	6	1	
	brigatinib	2	-	
	- no. of patients ^5^	29/30 (97%)	7/9 (78%)	
	- no. of patients with CBDP	15/30	4/7	
ALK TKI treatment, all lines (1–8)				
	crizotinib	23	9	19
	ceritinib	12	9	5
	alectinib	14	4	10
	brigatinib	4	0	3
	lorlatinib	3	1	1
	- no. of patients ^5,6^	29/30 (97%)	9/9 (100%)	22/23 (96%)
Chemotherapy, all lines (1–8)				
	platin-doublets	15	8	7
	monotherapy	6	4	6
	- no. of patients	14/30 (47%)	8/9 (89%)	8/23 (35%)
Summary of the complete treatment				
no. of treatment lines (mean; SD)		3.0; 1.5	4.0; 1.7	2.4; 1.6
no. of TKI treatment lines (mean; SD)		1.9; 1.2	2.6; 1.0	1.7; 1.1
patients with additional radiotherapy		18/30 (60%)	6/9 (67%)	12/21 (57%)
patients with additional surgical treatment ^7^		5/30 (17%)	1/9 (11%)	5/21 (24%)
Follow-up in months (median (25th–75th percentile))		36 (28–94)		

*TP53*wt^bas^: *TP53* wild-type at baseline; *TP53*wt^progr^: *TP53* wild-type at baseline and after disease progression; *TP53*mut^conv^: *TP53* wild-type at baseline with detection of *TP53* mutations at progression; *TP53*mut^bas^: *TP53* mutated at baseline; IQR: interquartile range; neg: negative; SD: standard deviation; PS: performance status; BL: baseline; PD: disease progression; no.: number; CBDP: continuation of treatment beyond disease progression due to ongoing clinical benefit; * *p* < 0.05 compared to *TP53*mut^conv^ and *p* < 0.05 compared to *TP53*mut^bas^. ^1^ 1/30 *TP53*wt^progr^ patients had an *EML4-ALK* V2 (E20;A20)^+^ large-cell neuroendocrine lung carcinoma. ^2^ The *ALK* fusion could be typed in 53/62 cases. ^3^ For 3/30 *TP53*wt^progr^ cases, *TP53* wild-type status at progression was evaluated by analysis of ctDNA samples obtained 24, 29 and 37 months later. ^4^ For 7/8 *TP53*wt^progr^ cases, also ctDNA at PD (neg); for 5/6 *TP53*wt^progr^ cases, also ctDNA at PD (neg). ^5^ One *TP53*wt^progr^ patient received definitive local treatment for oligometastatic disease and is still in remission without exposure to TKI; 2/9 *TP53*mut^conv^ patients did not receive next-line treatment after reassessment of *TP53* status due to rapid clinical deterioration (they had received TKI in previous lines). ^6^ One *TP53*mut^bas^ patient has ongoing stable disease 18 months after first-line chemotherapy without initiation of next-line treatment. ^7^ Excluding video-assisted thoracoscopy and pleurodesis for pleural effusion.

## Supplementary Materials

The following are available online at http://www.mdpi.com/2072-6694/11/1/124/s1, Figure S1: Progression-free survival of patients with metastatic ALK+ NSCLC under treatment with chemotherapy according to TP53 status at baseline and under therapy; Figure S2: Overall survival of study patients from initial diagnosis; Table S1: TP53 mutations of the study patients; Supplementary Method S1: RNA- and DNA-next-generation sequencing (NGS); Method S2: RT-PCR; Method S3: fluorescence in situ hybridisation (FISH); Method S4: ctDNA analysis.

## Abbreviations

ALK	anaplastic lymphoma kinase
NSCLC	non-small cell lung cancer
TKI	tyrosine kinase inhibitor
TNT TP53	time-to-next-treatment tumor protein p53
PFS	progression-free survival
OS	overall survival
