# Supplementary material for: Detection of TP53 Mutations in Tissue or Liquid Rebiopsies at Progression Identifies ALK+ Lung Cancer Patients with Poor Survival
Source: Cancers (Basel). 2019 Jan 21;11(1):124. doi: 10.3390/cancers11010124 (PMC6356563; doi:10.3390/cancers11010124)
Supplement: Supplementary file 1 [file cancers-11-00124-s001.pdf]

Supplementary Materials

Method S1. Diagnosis of NSCLC and detection of *EML4-ALK* fusion variants and *TP53* mutations by next-generation sequencing

The histological diagnosis of NSCLC and the quantification of tumour cell content were performed by experienced pulmonary pathologists on formalin-fixed and paraffin-embedded (FFPE) small biopsies according to the WHO and IASLC/ATS/ERS criteria [1]. Newly diagnosed cases were screened for the presence of an *ALK* alteration by fluorescence in situ hybridisation (FISH) and reverse-transcription polymerase-chain reaction (RT-PCR) until 2015, or by immunohistochemistry (IHC) and next-generation sequencing (NGS) afterwards, with FISH restricted to discordant results and technical failures.

For the NGS detection of *ALK* fusions and *TP53* mutations, areas with at least 15% tumour cellularity were marked on a hematoxylin and eosin-stained slide and macrodissected manually from consequent 8 µm thick tissue sections, followed by sample deparaffinization, digestion with Proteinase K overnight and nucleic acid extraction with the automated system Promega Maxwell 16 LEV RNA FFPE Purification Kit (Promega, Madison, WI, USA). Samples with at least 1 ng/µL DNA and/or RNA (QuBit 2.0 DNA and RNA high sensitivity kit, ThermoFisher Scientific, Waltham, MA, USA) were split into two and used for DNA and/or RNA library preparation directly and/or after treatment with DNaseI at room temperature for 15 min, respectively. For library preparation, the multiplex PCR-based Ion Torrent AmpliSeq™ technology (ThermoFisher) was used together with the RNA Lung Cancer Fusion Panel and a proprietary Lung Cancer Panel (covering the entire exons 4, 5, 6, 7, 8, 9, and 10 of *TP53* including 15 flanking base pairs for each exon), as described previously [2]. Only samples achieving library concentrations > 100 pM were considered suitable for further processing. Raw sequencing data were processed using the Torrent Suite Software (version 4.2.1) and aligned against the human genome (version hg19) using the Torrent Mapping Alignment Program algorithm. *ALK* fusion transcripts were detected using the fusion workflow integrated in the Ion Reporter Software (versions 4.2 and 5.2) as described previously [2].

Method S2. Detection of the V1 and V3 *EML4-ALK* fusion variants by RT-PCR

For the RT-PCR based detection of V1 and V3 *ALK* fusion variants, tumour RNA was reverse transcribed using the SuperScript III One Step RT-PCR System (Invitrogen, ThermoFisher) and amplified with primers specific for the *EML4* exons 6 and 13 and the *ALK* exon 20 (sequences given in the Online Supplements). Direct sequencing of the PCR amplicons was performed for both strands on a 3500 Genetic Analyzer using the BigDye Terminator v1.1 Cycle Sequencing Kit (both from Applied Biosystems, Life Technologies, Carlsbad, CA, USA). Since RT-PCR represented only a temporary

solution in our laboratory and was succeeded by NGS in 2015 for the typing of *ALK* fusion variants, we chose not to invest additional resources in the establishment of RT-PCR assays specific for less frequent variants like V2.

Method S3. ALK IHC and FISH

For the ALK IHC a sensitive and specific antibody (D5F3 clone, Roche, Mannheim, Germany) [3] was used according to current guidelines [4]. *ALK* FISH analyses were performed on whole block slides of FFPE NSCLC samples using a break-apart probe (ZytoLight SPEC ALK probe, ZytoVision GmbH, Bremerhaven, Germany). At least 100 cells were analysed per case, and cases were considered to be positive when 15% or more of 50 tumour cells showed classic split signals [4].

Method S4. ctDNA analysis

For ctDNA analysis, plasma was isolated from blood samples of ALK^+ NSCLC patients centrifuged within 30 minutes of collection and processed with the AVENIO ctDNA Analysis Kit (covering the entire *TP53* exons 2, 3, 4, 5, 6, 7, 8, 9, 10, 11) according to the manufacturer's instructions (Roche Diagnostics, Mannheim, Germany). Briefly, DNA was isolated from 2 mL of plasma using the AVENIO cfDNA Isolation Kit (Roche) and quantified with the Qubit dsDNA High Sensitivity Kit (ThermoFisher). Targeted sequencing libraries were prepared from 39.5 ng DNA in median (range 15–50 ng) using the AVENIO ctDNA Library Preparation Kit with the AVENIO Targeted Panel (both from Roche) for hybridisation-based enrichment of a 17-gene panel. All protocols were conducted according to the manufacturer's recommendations. Equal amounts of 16 libraries were pooled and sequenced on an Illumina NextSeq 550 using the High Output Kit V2 (300 cycles) according to the manufacturer's protocol (Illumina, San Diego, CA, USA) with a median unique target sequence coverage of 7,600x (range 2,013×–12,467×). Automated raw data processing and data analysis was performed with the AVENIO ctDNA analysis software (Roche).

Table S1. *TP53* mutations of the study patients.

ALK fusion variant	*TP53* status	*TP53* mutation	Material of 1st detection	*TP53* exon affected	Effect
V1 (E13;A20)	*TP53*mut^{conv}	p.?: c.994-2A>G; AF 0.24% (ctDNA); CV: 5446	ctDNA	loss of exon 10	Loss-of-function[1,2]
V1 (E13;A20)	*TP53*mut^{conv}	p.H214R: c.641A>G; AF: 0.07% (ctDNA): 12103	ctDNA	6	Loss-of-function[2]
V2 (E20;A20)	*TP53*mut^{conv}	p.K139E: c.415A>G; AF: 0.25% (ctDNA); 7158	ctDNA	5	Loss-of-function[2]
V3 (E6;A20)	*TP53*mut^{conv}	p.E285K: c.853G>A; AF: 11%; CV: 2506	FFPE	8	Likely loss-of-function[1]
V3 (E6;A20)	*TP53*mut^{conv}	p.Y234fs*8:c.699_700insCATCCAC; AF:26%; CV:2918	FFPE	7	Loss-of-function[2]
V3 (E6;A20)	*TP53*mut^{conv}	p.M237I: c.711G>A; AF: 0.18% (ctDNA); CV: 8496	ctDNA	7	Loss-of-function[2]
V3 (E6;A20)	*TP53*mut^{conv}	p.R158S: c.472C>A; AF: 0.09% (ctDNA); CV: 5487	ctDNA	5	Loss-of-function[1]
V3 (E6;A20)	*TP53*mut^{conv}	p.A276D: c.827C>A; AF: 0.64% (ctDNA); CV: 5657	ctDNA	8	Loss-of-function[1]
K9;A20	*TP53*mut^{conv}	p.I195Y: c.584T>C; AF 0.16% (ctDNA), CV: 6290<br>p.V216L: c.646G>T; AF 0.15% (ctDNA), CV: 7542	ctDNA	6	Loss-of-function[2]
E9;A20	*TP53*mut^{bas}	p.S241C: c.722C>G; AF 0.17% (ctDNA); CV: 5147	FFPE	7	Loss-of-function[2]
V1 (E13;A20)	*TP53*mut^{bas}	p.L194P:c.581T>C; AF 14%, CV: 277	FFPE	6	Loss-of-function[2]
V1 (E13;A20)	*TP53*mut^{bas}	p.R342P: c.1025G>C; AF 30%, CV: 900	FFPE	10	Loss-of-function[1]
V1 (E13;A20)	*TP53*mut^{bas}	p.spl?: c.375+1G>A; AF 14%, CV: 2639	FFPE	likely loss of exon 5	Likely loss-of-function[2,3]
V1 (E13;A20)	*TP53*mut^{bas}	p.L330fs*15: c.988delC; AF 35%, CV: 1996	FFPE	9	Loss-of-function[1]
V1 (E13;A20)	*TP53*mut^{bas}	p.V272M: c.814G>A; AF: 23%, CV: 13056	FFPE	8	Loss-of-function[1]
V1 (E13;A20)	*TP53*mut^{bas}	p.S183*: c.548C>G; AF: 24%, CV: 883	FFPE	5	Loss-of-function[1]

V2 (E20;A20)	*TP53*mut^bas^	p.R273C: c.817C>T; AF: 23%, CV: 6755	FFPE	8	Loss-of-function[1]
V2 (E20;A20)	*TP53*mut^bas^	p.P152S: c.454C>T; AF 26%, CV: 971	FFPE	5	Loss-of-function[1]
V2 (E20;A20)	*TP53*mut^bas^	p. H193D: c.577C>G; AF 54%, CV: 530	FFPE	6	Loss-of-function[1]
V3 (E6;A20)	*TP53*mut^bas^	p.R249W: c.745A>T; AF 45%, CV: 1120	FFPE	7	Loss-of-function[1]
V3 (E6;A20)	*TP53*mut^bas^	p.M66fs*84: c.195_196insCAGA; AF:33%, CV: 1735	FFPE	4	Likely loss-of-function[4]
V3 (E6;A20)	*TP53*mut^bas^	p.E286delinsDRRTEE: c.857_858insCCGGCGCACAGAGGA; AF: 20%, CV: 1914	FFPE	8	Likely loss-of-function[1]
V3 (E6;A20)	*TP53*mut^bas^	p.R213Q: c.638G>A; AF: 11%, CV:2215	FFPE	6	Loss-of-function[1,2]
V3 (E6;A20)	*TP53*mut^bas^	p.Q331*: c.991C>T; AF:10%, CV: 1591<br>p.C135F: c.404G>T; AF:12%, CV: 1566	FFPE	9<br>5	Loss-of-function[1,2]<br>Loss-of-function[1,2,4]
V3 (E6;A20)	*TP53*mut^bas^	p.spl?: c.920-2A>G, AF:13%, CV: 1676	FFPE	10	Likely loss-of-function[2,3]
V3 (E6;A20)	*TP53*mut^bas^	p.Y220C: c.659A>G, AF: 0.19% (ctDNA), CV: 2146	ctDNA [5]	6	Loss-of-function[1]
V3 (E6;A20)	*TP53*mut^bas^	p.Q192*: c.574C>T, AF: 13%, CV: 308	FFPE	6	Loss-of-function[1]
V9 (E18;A20)	*TP53*mut^bas^	p.Y220C: c.659A>G; AF: 15%, CV 607	FFPE	6	Loss-of-function[2]
V9 (E18;A20)	*TP53*mut^bas^	p.Leu167fs*41: c.508_508A>delinsGG; AF: 24%, CV: 1445	FFPE	5	Loss-of-function[2]
unknown	*TP53*mut^bas^	p.R249W: c.745A>T; AF: 17%, CV: 4162	FFPE	7	Loss-of-function[1]
unknown	*TP53*mut^bas^	p.A138V: c.413C>T; AF: 20%, CV: 2889	FFPE	5	Loss-of-function[2]
unknown	*TP53*mut^bas^	p.R110L: c.329G>T; AF13%, CV: 352	FFPE	4	Likely loss-of-function[1]

AF: allelic frequency; CV: coverage; *TP53*mut^{conv}: cases with wild-type *TP53* at baseline and detection of *TP53* mutations at disease progression ; *TP53*mut^{bas}: cases with detection of *TP53* mutations at baseline.

[1] according to the OncoKB database, http://oncokb.org/#/gene/TP53, accessed on 04.04.2018 [5].

[2] according to the COSMIC database v84, released 13 Feb 2018, https://cancer.sanger.ac.uk/cosmic/.

[3] according to the ClinVar database, https://www.ncbi.nlm.nih.gov/clinvar/, accessed on 04.04.2018.

[4] according to the The Jackson Laboratory database, https://ckb.jax.org/gene/show?geneId=7157, accessed on 04.04.2018.

[5] for this single case, the *TP53* mutation at baseline was detectable with ctDNA analysis only, while the FFPE tumour sample was negative.

Figure S1. Progression-free survival of patients with metastatic ALK+ NSCLC under treatment with chemotherapy according to *TP53* status at baseline and under therapy. The median progression-free survival (PFS) under chemotherapy treatment was 5 months for patients with *TP53* mutations at baseline (*TP53*mut^{bas}) vs. 7 months for patients with initially wild-type status and detection of TP53 mutations in a subsequent biopsy (*TP53*mut^{conv}) vs. 8 months for patients without subsequent detection of *TP53* mutations (*TP53*wt^{progr}, logrank $p = 0.60$). The treatment details are given in Table 1; ns: not statistically significant.

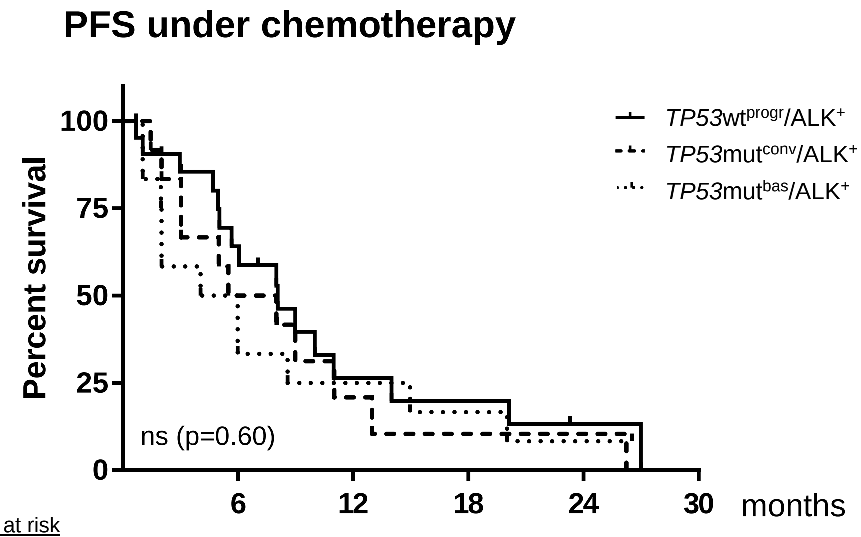

No. at risk

*TP53*wt^{progr}:	21	11	4	3	1	0
TP53mut^{conv}:	12	6	2	1	1	0
*TP53*mut^{bas}:	13	4	3	2	1	0

Figure S2. Overall survival of study patients from initial diagnosis. The median overall survival (OS) from initial diagnosis was 44 months for patients with *TP53* mutations at baseline (*TP53*mut^{bas}) vs. 45 months for patients with initially wild-type status and detection of TP53 mutations in a subsequent biopsy (*TP53*mut^{conv}) vs. not reached for patients without subsequent detection of *TP53* mutations (*TP53*wt^{progr}, logrank p=0.0012). As shown in Table 1, 10/30 *TP53*wt^{progr} and 2/23 *TP53*mut^{bas} patients had secondary development of metastatic disease after relapse of initially nonmetastatic ALK^{+} NSCLC, causing the curves in this plot to differ slightly from these of Figure 1; ns: not statistically significant.

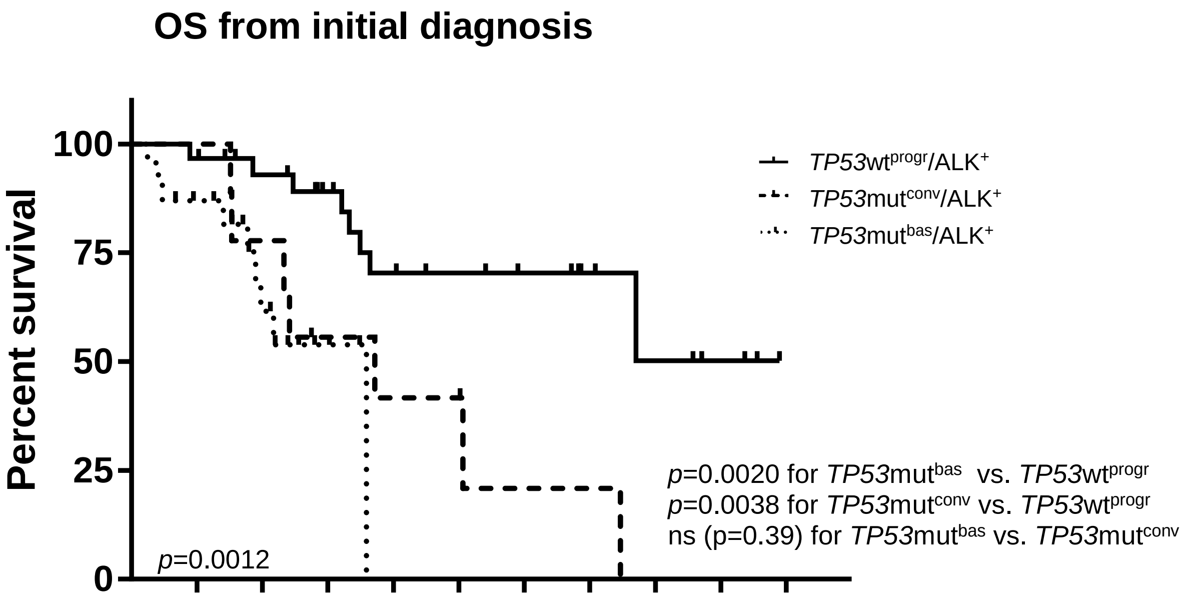

No. at risk		1	2	3	4	5	6	7	8	9	10
*TP53*wt^{progr}:	30	29	25	20	15	13	11	8	5	3	0
*TP53*mut^{conv}:	9	9	7	4	3	3	1	1	0		
*TP53*mut^{bas}:	23	17	9	3	0						

References

1. Travis, W.D.; Brambilla, E.; Noguchi, M.; Nicholson, A.G.; Geisinger, K.; Yatabe, Y.; Ishikawa, Y.; Wistuba, I.; Flieder, D.B.; Franklin, W., *et al.* Diagnosis of lung cancer in small biopsies and cytology: Implications of the 2011 International Association for the Study of Lung Cancer/American Thoracic Society/European Respiratory Society classification. *Arch Pathol Lab Med* **2013**, *137*, 668-684.
2. Pfarr, N.; Stenzinger, A.; Penzel, R.; Warth, A.; Dienemann, H.; Schirmacher, P.; Weichert, W.; Endris, V. High-throughput diagnostic profiling of clinically actionable gene fusions in lung cancer. *Genes Chromosomes Cancer* **2016**, *55*, 30-44.
3. Mino-Kenudson, M.; Chirieac, L.R.; Law, K.; Hornick, J.L.; Lindeman, N.; Mark, E.J.; Cohen, D.W.; Johnson, B.E.; Janne, P.A.; Iafrate, A.J., *et al.* A novel, highly sensitive antibody allows for the routine detection of ALK-rearranged lung adenocarcinomas by standard immunohistochemistry. *Clin Cancer Res* **2010**, *16*, 1561-1571.
4. Lindeman, N.I.; Cagle, P.T.; Beasley, M.B.; Chitale, D.A.; Dacic, S.; Giaccone, G.; Jenkins, R.B.; Kwiatkowski, D.J.; Saldivar, J.S.; Squire, J., *et al.* Molecular testing guideline for selection of lung cancer patients for EGFR and ALK tyrosine kinase inhibitors: Guideline from the college of american pathologists, international association for the study of lung cancer, and association for molecular pathology. *J Thorac Oncol* **2013**, *8*, 823-859.
5. Chakravarty, D.; Gao, J.; Phillips, S.M.; Kundra, R.; Zhang, H.; Wang, J.; Rudolph, J.E.; Yaeger, R.; Soumerai, T.; Nissan, M.H., *et al.* Oncokb: A precision oncology knowledge base. *JCO Precis Oncol* **2017**, *2017*.
